# Claudin-9 constitutes tight junctions of folliculo-stellate cells in the anterior pituitary gland

**DOI:** 10.1038/s41598-021-01004-z

**Published:** 2021-11-04

**Authors:** Atsuko Y. Higashi, Tomohito Higashi, Kyoko Furuse, Kana Ozeki, Mikio Furuse, Hideki Chiba

**Affiliations:** 1 Department of Basic Pathology, Fukushima Medical University, 1 Hikariga-oka, Fukushima, 960-1295 Japan; 2 Department of Nephrology and Hypertension, Fukushima Medical University, Fukushima, 960-1295 Japan; 3 Division of Cell Structure, National Institute for Physiological Sciences, Okazaki, Aichi 444-8787 Japan; 4 Department of Physiological Sciences, School of Life Science, SOKENDAI (Graduate University for Advanced Studies), Okazaki, Aichi 444-8585 Japan

**Keywords:** Tight junctions, Pituitary gland

## Abstract

The anterior pituitary gland regulates growth, metabolism, and reproduction by secreting hormones. Folliculo-stellate (FS) cells are non-endocrine cells located among hormone-producing cells in the anterior pituitary glands. They form follicular lumens, which are sealed by tight junctions (TJs). Although FS cells are hypothesized to contribute to fine-tuning of endocrine cells, little is known about the exact roles of FS cells. Here, we investigated the molecular composition of TJs in FS cells. We demonstrated that occludin is a good marker for TJs in the pituitary gland and examined the structure of the lumens surrounded by FS cells. We also found that claudin-9 is a major component of TJs in the FS cells. In immunoelectron microscopy, claudin-9 was specifically localized at TJs of the FS cells. The expression of claudin-9 was gradually increased in the pituitary gland after birth, suggesting that claudin-9 is developmentally regulated and performs some specific functions on the paracellular barrier of follicles in the pituitary gland. Furthermore, we found that angulin-1, angulin-2, and tricellulin are localized at the tricellular contacts of the FS cells. Our findings provide a first comprehensive molecular profile of TJs in the FS cells, and may lead us towards unveiling the FS cell functions.

## Introduction

**Figure 1:**
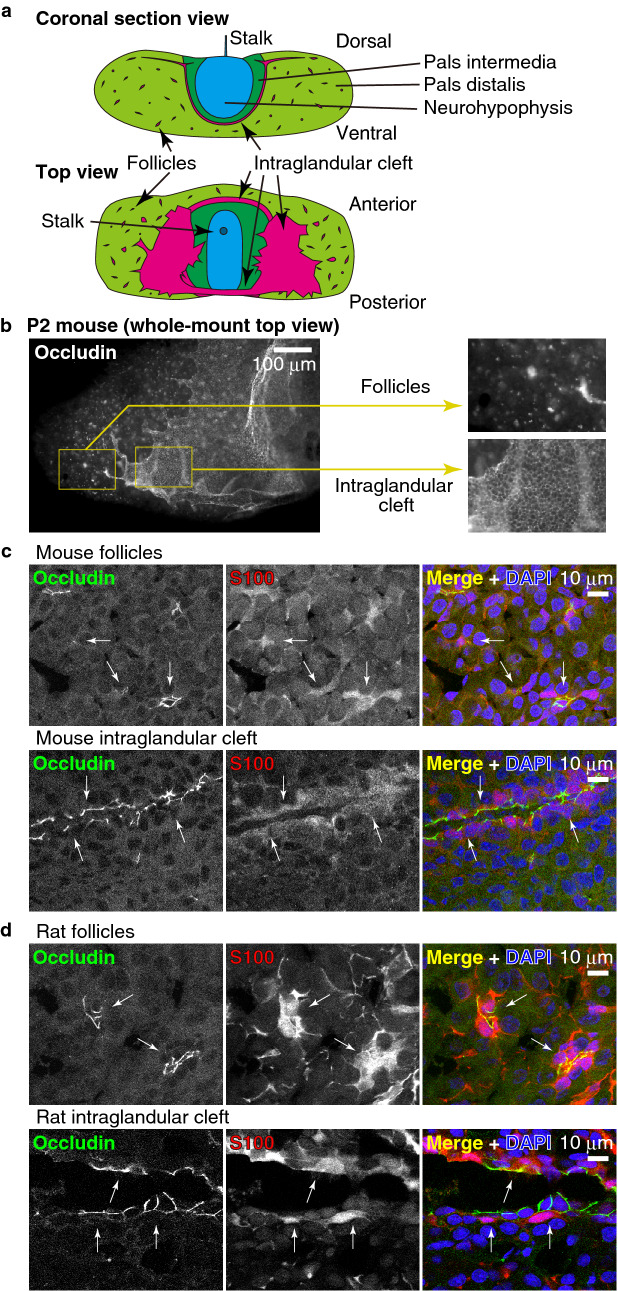
Anterior pituitary gland and FS cells. (**a**) Schematic illustration of the mouse pituitary gland. (**b**) Whole-mount staining of the pituitary gland of a mouse neonate (P2) using anti-occludin mAb. (**c**), (**d**) Immunostaining of frozen sections of the adult mouse (**c**) or adult rat (**d**) anterior pituitary gland using anti-occludin mAb, anti-S100 pAb, and DAPI.

The anterior pituitary gland (also known as adenohypophysis) is an endocrine organ that secretes several hormones to regulate growth, metabolism, reproduction, and stress responses. Histologically, the anterior pituitary gland is composed of three parts, the pars distalis (pars anterior), pars intermedia, and pars tuberalis (Fig. 1a). The pars distalis and pars intermedia are separated by the intraglandular cleft (Fig. 1a), which is a residual lumen of Rathke's pouch, an embryological structure arising from the primitive oral cavity. Hormone-secreting cells are chromophilic and are classified into five cell types: somatotroph (α-acidophil, which secretes growth hormones [GH]), thyrotroph (β-basophil, which secretes thyroid-stimulating hormone [TSH]), gonadotroph (δ-basophil, which secretes follicle-stimulating hormone [FSH] and luteinizing hormone [LH]), lactotroph (ε-acidophil, which secretes prolactin [PRL]) and corticotroph (secretes proopiomelanocortin [POMC]-derived hormones such as adrenocorticotrophic hormone [ACTH] and β-endorphin). In addition to the hormone-secreting cells, the anterior pituitary glands contain agranular chromophobic cells known as folliculo-stellate cells (FS cells), which were first discovered in 1953^1^. FS cells cover the luminal surface of the intraglandular cleft, or make small clusters to form follicles or ductiles, which contain low-electron-density colloids^2^. FS cells in the clusters have a star-shaped morphology and are interconnected to each other through their long processes to make a network in the anterior pituitary gland^1–3^. FS cells express S100 protein^4,5^ and β-adrenergic receptor^6^. Although many roles have been suggested for FS cells (reviewed in^3,7–10^), including ACTH production^2,11^, waste scavenging^12,13^, ammonia removal^14^, mechanical support^13,15^, transport of nutrients from blood vessels^9,16^, electrophysiological communication^17^, and a cell source of hormone-secreting cells^18–23^, the precise functions of FS cells remain elusive.

Transmission electron microscopic studies have revealed that the follicles surrounded by FS cells have an apical junctional complex at the cell–cell interfaces of FS cells in various species, such as human^24^ and rats^25^. The apical junctional complex is found in most epithelial cell types and is composed of tight junctions (TJs, also known as *zonula occludens*), adherens junctions (AJs, also known as *zonula adhaerens*), and desmosomes (also known as *macula adhaerens*)^26^. AJs and desmosomes contribute to the mechanical strength and tissue integrity of the epithelial sheet and TJs are responsible for maintenance of epithelial barrier function. In AJs, transmembrane protein cadherins make an intercellular bridge through the extracellular domain, and make a mechanical connection to the cytoskeleton with the intracellular tail through cytoplasmic plaque proteins α- and β-catenin. In FS cells, E-cadherin, a major cadherin expressed in epithelial cells, is expressed and localized at cell–cell interfaces^27^. TJs are composed of small four-transmembrane domain protein claudins, which polymerize within the plasma membrane and form a tight seal between adjacent cells through transcellular interaction by minimizing the paracellular space^28–31^. Claudins constitute a multi-gene family with ~ 24 members and exhibit tissue-specific expression patterns^32^. It is believed that the combination of expressed claudins determines the physiological characteristics of paracellular permeability of the tissues. Another four-transmembrane domain protein occludin^33^ and the cytoplasmic plaque protein ZO-1^34^ are also major components of TJs in epithelial cells. Tricellular tight junctions (tTJs) are specialized TJ structures at the vertices of epithelial cells^35,36^ serving as a barrier at three cell junctions. The occludin-related protein tricellulin and immunoglobulin superfamily protein angulins are known components of tTJ in most epithelial cells^37,38^. To date, there have been no comprehensive studies on the expression profiles and localization of TJ and tTJ proteins in the pituitary gland.

Here, we investigated the molecular composition of TJs and tTJs in FS cells by the quantitative reverse transcriptase-polymerase chain reaction (qRT-PCR), immunostaining, immunoprecipitation and immunoelectron microscopy, and found that claudin-9 is specifically localized at the TJs of FS cells.

## Results

### Occludin is localized at the cell–cell junctions of the follicles surrounded by FS cells

**Figure 2:**
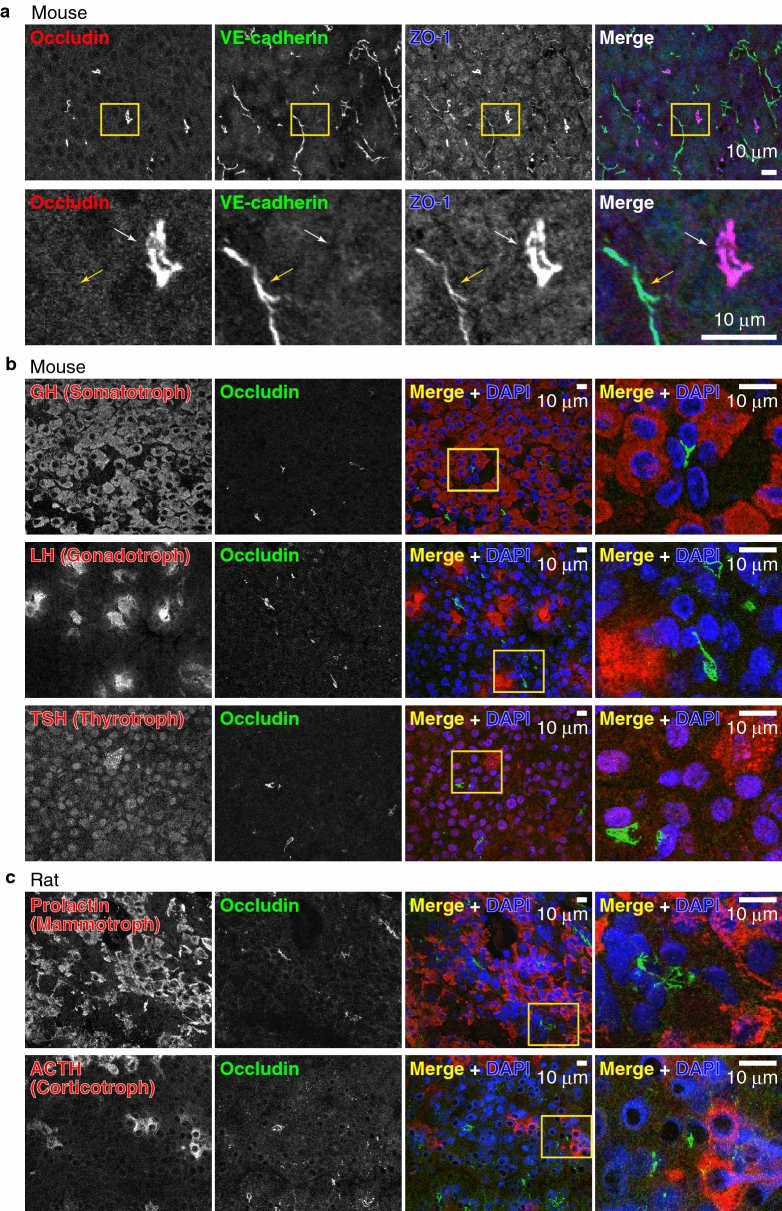
Occludin-positive cells are FS cells. (**a**) Triple-staining of the adult mouse anterior pituitary gland using anti-occludin mAb, anti-VE-cadherin pAb and anti-ZO-1 pAb. Note that occludin stains the ZO-1-positive FS cells (white arrows) and does not stain the endothelial cells labeled with both VE-cadherin and ZO-1 (yellow arrows). (**b**), (**c**) Immunostaining of the adult mouse (**b**) or rat (**c**) anterior pituitary gland using anti-GH, LH, TSH, Prolactin or ACTH pAbs and anti-occludin mAb. Note that occludin does not colocalize with any hormone signals.

To examine whether the general epithelial TJ marker protein occludin is expressed in the FS cells and localized at the TJs, we immunostained the pituitary glands of a postnatal day (P) 2 mouse with anti-occludin antibody (Fig. 1b). Occludin signals were clearly detected in the follicles and intraglandular clefts (Fig. 1b). The small lumens of the follicles are discretely distributed and are independent of the lumen of the intraglandular cleft. A similar structure was observed in whole-mount staining with anti-ZO-1 in adult mice (see Supplementary Fig. S1 online). To confirm that occludin is expressed in FS cells, we stained frozen sections of the anterior pituitary glands of mice with an anti-occludin antibody and the FS cell marker S100 (Fig. 1c). Occludin was detected at the cell–cell junctions between epithelial cells delineating the intraglandular cleft of the glands (Fig. 1c). These epithelial cells are considered FS cells because they are S100-positive and extend long processes between other hormone-producing cells. We also examined the anterior pituitary glands of rats and obtained similar results (Fig. 1d). Since it has been reported that occludin is also expressed in the endothelial TJs in the central nervous system^33^, we next examined whether the occludin-positive cells include endothelial cells of blood vessels. We triple-stained the mouse pituitary gland with anti-occludin monoclonal antibody (mAb) together with anti-VE-cadherin (an endothelial junction marker), and anti-ZO-1 (a general cell–cell junction marker) antibodies. As shown in Fig. 2a, occludin was detected only at the junctions of the FS cells and was not detected at the endothelial junctions stained with VE-cadherin and ZO-1. Cells with occludin-positive follicles were not stained with anti-GH, anti-LH, anti-TSH, anti-Prolactin, or anti-ACTH antibodies (Fig. 2b, c), further supporting that occludin-positive cells are FS cells. These results indicate that occludin is expressed in FS cells and is specifically localized at cell–cell junctions of FS cells. Hence, we use occludin as a marker for the cell–cell junctions of FS cells.

### Claudin-9 is abundantly expressed in the pituitary gland

**Figure 3:**
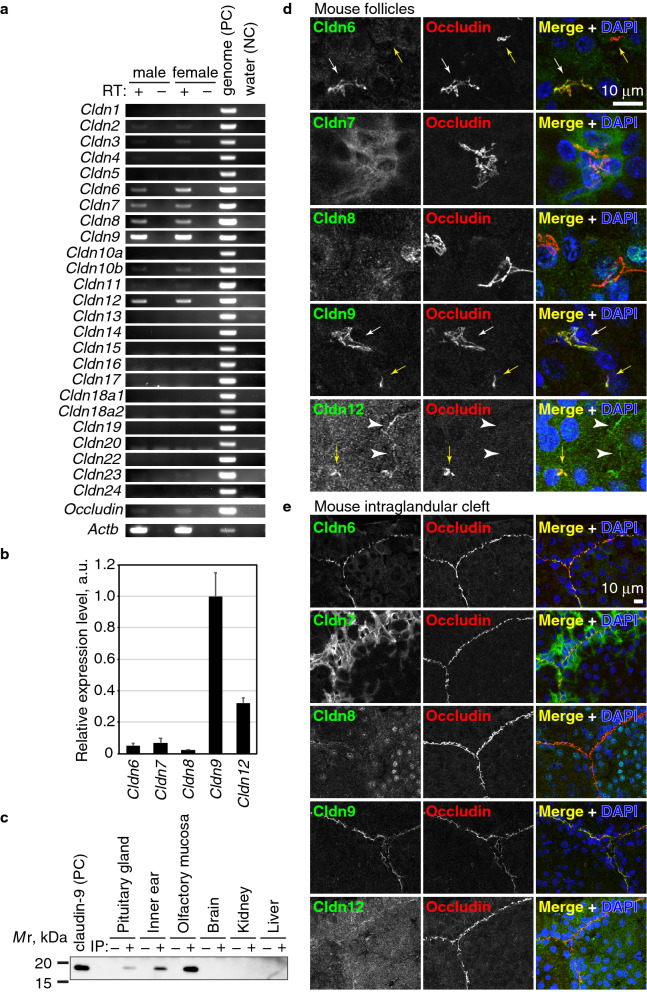
Claudin-9 is a component of FS cells. (**a**) RT-PCR of the pituitary gland of adult male and female mice. Since the forward and reverse primers were designed in the same exon, the genome DNA was used as a positive control (PC) and water was used as a negative control (NC). The reaction mix without reverse transcriptase (RT) also served as a negative control. (**b**) Real-time RT-PCR of the adult mouse pituitary gland. The expression levels were normalized using known concentration of DNA fragments as standards. The expression level of *cldn9* was set to 1. Error bars indicate s.d. (**c**) Immunoprecipitation of claudin-9 from adult mouse organs using rat anti-claudin-9 (clone 1B1) and immunoblotting using goat anti-claudin-9 pAb. Human CLDN9-expressing HEK293T cell lysate served as a positive control (PC). Note that the claudin-9 band was detected in the pituitary gland lysate as well as in the inner ear and olfactory mucosa. (**d**), (**e**) Immunostaining of the adult mouse anterior pituitary gland using anti-claudin-6 pAb, anti-claudin-7 pAb, anti-claudin-8 pAb, anti-claudin-9 mAb (clone 1B1), or anti-claudin-12 pAb together with anti-occludin mAb or pAb and DAPI.

Most epithelial cells express multiple claudins in a specific combination, which determines the ion- and charge-specific permeability of the paracellular pathway in epithelial tissues. To identify claudins expressed in the mouse pituitary gland, we performed comprehensive RT-PCR using specific primer pairs for each claudin (Fig. 3a). Since we designed each primer set within the same exon, we used mouse genome DNA as a positive control and samples without reverse transcription reaction (RT−) as a negative control. Among 25 mouse claudins, including splice isoforms of *cldn10* and *cldn18*, mRNAs for five claudins (*cldn6*, *cldn7*, *cldn8*, *cldn9*, and *cldn12)* were detected in the male and female pituitary glands (Fig. 3a). We quantified the relative expression levels of these five claudins by quantitative RT-PCR using PCR products as standard and statistical analysis showed that mRNAs for *cldn9* and *cldn12* were more abundantly expressed than other three claudins in the pituitary gland (Fig. 3b). The expression of *cldn9* was significantly higher than *cldn12*. To examine the protein expression of claudin-9, we performed immunoprecipitation. Since many antibodies against claudins frequently cross-react with other claudins, we established new rat mAbs against the cytoplasmic tail of claudin-9 using the iliac lymph node method^39^. The amino-acid sequence of claudin-9 used for immunization is shared by humans, mice and rats (see Supplementary Fig. S2 online). We obtained two clones with different epitopes, 1B1 and 1B8. Both clones successfully stained the cell–cell junctions of the sensory epithelium in the cochlea of the inner ear and olfactory receptor neurons in the olfactory epithelium (see Supplementary Fig. S2 online), which have been reported to express claudin-9^40–42^. These mAbs did not stain cell–cell junctions in other epithelial tissues including kidney, intestine, and liver (data not shown), indicating that they are specific for claudin-9. Next, to demonstrate that claudin-9 protein is expressed in the pituitary gland, we performed Western blot analysis using the antibodies. However, no significant band was obtained from the maximum amount of lysate applied in a gel lane, probably because of the low expression level (data not shown). Thus, we immunoprecipitated claudin-9 protein from the mouse pituitary gland lysate. We used the claudin-9-overexpressing HEK293T cell lysate, inner ear, and olfactory mucosa as positive controls, and the brain, kidney and liver as negative controls. As shown in Fig. 3c, the claudin-9 band was clearly detected in the pituitary gland, as well as in the inner ear and olfactory mucosa, but not in the brain, kidney, or liver, indicating that claudin-9 is specifically expressed in the pituitary gland.

### Claudin-9 is localized at cell–cell contacts of mouse FS cells

Next, we examined the localization of claudin-9 protein by immunofluorescent staining. Anti-claudin-9 mAb clone 1B1 stained the cell–cell junctions of FS cells in the follicles and intraglandular clefts in a pattern similar to that of occludin (Fig. 3d, e), suggesting that claudin-9 constitutes the TJs of FS cells. The other mAb clone 1B8 and the commercially available goat polyclonal antibody (pAb) also stained the cell–cell junctions of FS cells (see Supplementary Fig. S3 online). We also examined other claudins detected in the anterior pituitary gland by RT-PCR. Claudin-6 was also detected at cell–cell junctions of the follicles (Fig. 3d, white arrows) and intraglandular cleft (Fig. 3e), although a relatively small occludin-positive follicles had no claudin-6 signal (Fig. 3d, yellow arrows). Claudin-7 was detected at the lateral membranes of FS cells (Fig. 3d, e). Claudin-8 was hardly detected (Fig. 3d, e). Anti-claudin-12 pAb stained the cell–cell junctions of both follicular FS cells (Fig. 3d, yellow arrows) and endothelial cells (Fig. 3d, white arrowheads), and had faint signals at the cell–cell junctions of the FS cells in the intraglandular clefts (Fig. 3e). These data indicate that claudin-9 is a major component of cell–cell junctions in FS cells, and claudin-6 and claudin-12 may also be incorporated.

### Claudin-9 is localized at the TJs of FS cells

**Figure 4:**
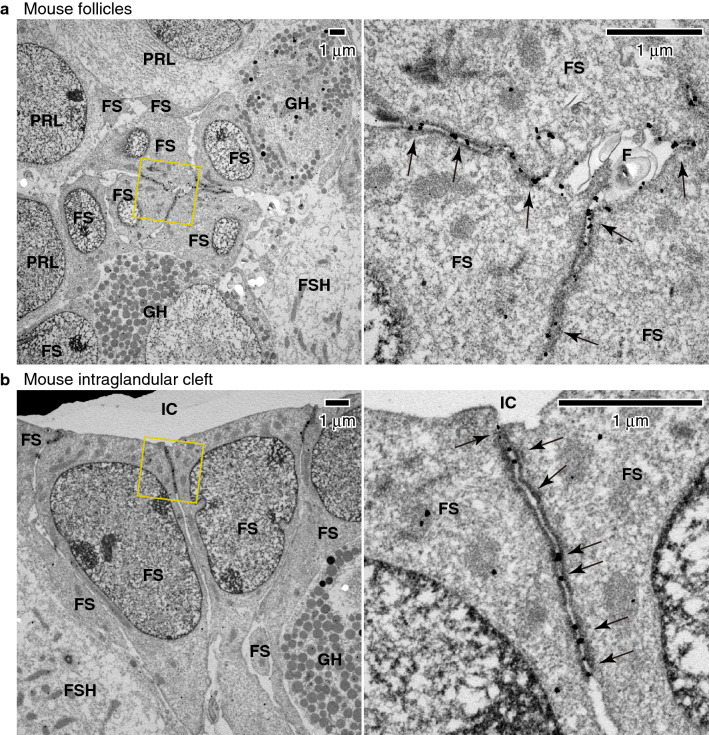
Claudin-9 is localized at the TJs of FS cells. (**a**,**b**) Immunoelectron microscopy of the adult mouse anterior pituitary gland. Claudin-9 signal was detected at TJs (arrows) between the FS cells (FS) in the follicles (F) (**a**) and the intraglandular cleft (IC) (**b**). FSH, FSH cells; PRL, PRL cells; GH, GH cells.

To further examine the localization of claudin-9, we performed immunoelectron microscopy of the mouse pituitary gland using goat anti-claudin-9 pAb. In electron microscopy, TJs were identified as an electron-dense membrane apposition structure at the most apical part of the cell–cell junctions of FS cells. The claudin-9 signal was clearly localized at TJs in the follicles (Fig. 4a) and intraglandular clefts (Fig. 4b), indicating that claudin-9 constitutes the TJs of FS cells.

### Claudin-9 is not expressed in the developing anterior pituitary gland

During embryogenesis, the anterior pituitary gland originates from the evagination of the stomadeum (the roof of the developing mouth). The evagination becomes a cyst called Rathke's pouch, which then makes contact with the infundibular process of the neuroectoderm, and differentiates into the anterior pituitary gland. We examined whether claudin-9 is expressed during development (see Supplementary Fig. S4 online). In the Rathke's pouch at E13.5, claudin-9 was not detected. Claudin-9 was expressed in the follicles and intraglandular clefts of E17.5 embryos, although only FS cells of the intraglandular clefts at the pars distalis were positive. The FS cells of the intraglandular clefts in the pars intermedia became positive for claudin-9 between P0 and P7. Occludin was expressed and localized at TJs of FS cells throughout the process of development. These data indicate that claudin-9 becomes expressed after differentiation of the anterior pituitary gland.

### Tricellulin, angulin-1, and angulin-2 are localized at the tricellular contacts of FS cells

**Figure 5:**
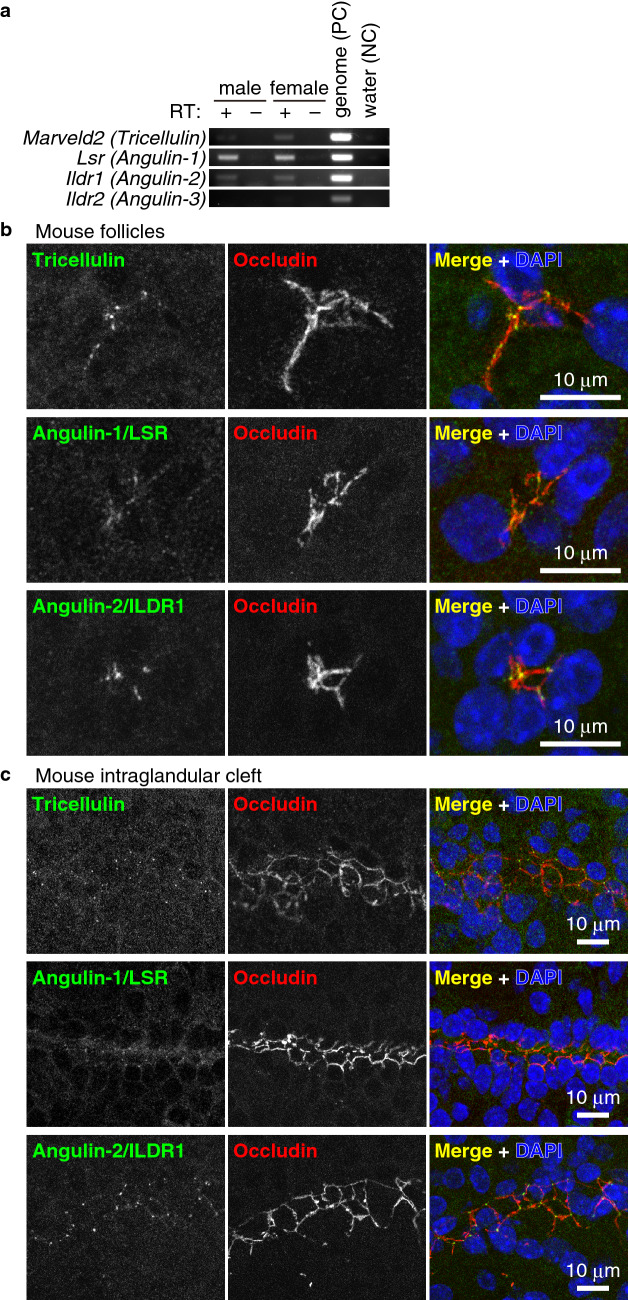
Tricellulin, angulin-1, and angulin-2 are expressed in FS cells. (**a**) RT-PCR of the pituitary gland of adult male and female mice. (**b**), (**c**) Immunostaining of the adult mouse anterior pituitary gland using anti-tricellulin mAb (green) and anti-occludin pAb (red), anti-angulin-1 pAb (green) and anti-occludin mAb (red), and anti-angulin-2 pAb (green) and anti-occludin mAb (red) together with DAPI (blue).

We also examined the expression and localization of components of tTJs, tricellulin and angulins, in the FS cells. Angulins are composed of three members, angulin-1 (also known as lipolysis-stimulated lipoprotein receptor [LSR]), angulin-2 (also known as immunoglobulin-like domain containing receptor 1 [ILDR1]), and angulin-3 (also known as ILDR2). RT-PCR revealed that mRNAs for tricellulin, angulin-1, and angulin-2 were expressed in the mouse pituitary glands (Fig. 5a). Immunostaining of frozen sections showed that tricellulin, angulin-1 and angulin-2 proteins were expressed in the follicles and intraglandular clefts of mouse anterior pituitary glands and that they were localized at the tricellular vertices of FS cell junctions labeled with occludin (Fig. 5b, c). In spite of having a lower expression level compared with angulin-1, angulin-2 had stronger and more concentrated signals at the tricellular contacts of FS cells, suggesting that angulin-2 is more preferentially localized at tricellular contacts compared with angulin-1 when both proteins are expressed in the same cell^43^. These data suggest that tTJs in FS cells are composed of tricellulin, angulin-1, and angulin-2.

### Claudin-9 is expressed in human FS cells

**Figure 6:**
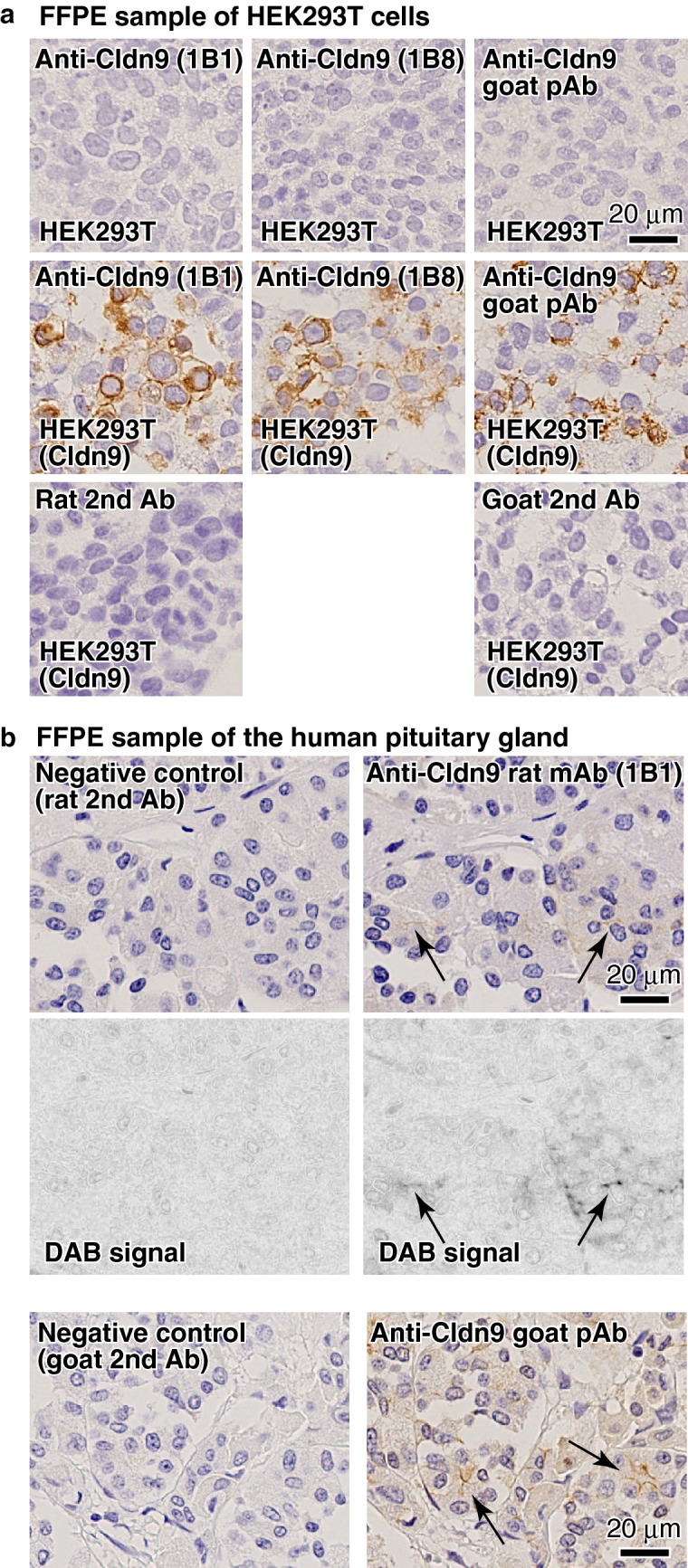
Anti-claudin-9 is applicable for immunohistochemistry. (**a**) Immunocytochemical staining of FFPE samples of HEK293T cells expressing human CLDN9 using anti-claudin-9 mAbs (clones 1B1 and 1B8) and anti-claudin-9 pAb. (**b**) Immunohistochemical staining of FFPE samples of the human pituitary gland using anti-claudin-9 mAb (clone 1B1) and anti-claudin-9 pAb.

Finally, we tested the expression of claudin-9 in human FS cells. We examined whether the newly established rat anti-claudin-9 mAbs and commercial goat anti-claudin-9 pAb are applicable in immunohistochemistry (IHC) using formalin-fixed paraffin-embedded (FFPE) samples of HEK293T cells transiently overexpressing human claudin-9 (Fig. 6a). Both rat anti-claudin-9 mAbs and goat anti-claudin-9 pAb strongly stained HEK293T cells overexpressing claudin-9, but not the parental HEK293T cells. There was no staining when primary antibodies were omitted (Fig. 6a). Then, using rat anti-claudin-9 mAb (clone 1B1) and goat anti-claudin-9 pAb, we immunostained the FFPE sample of the human pituitary gland. Both antibodies exhibited cell–cell junction patterns in the anterior pituitary glands, and there was no signal without primary antibodies (Fig. 6b), suggesting that claudin-9 is also a component of cell–cell junctions in human FS cells.

## Discussion

In the present study, we investigated the structure of the lumens of FS cells in the anterior pituitary gland and found that the small discrete lumens of the follicles were distributed throughout the tissue. Although FS cells form an interconnected network throughout the anterior pituitary gland, the follicular lumens are separated from each other and the intraglandular cleft lumen. We showed that claudin-9 is abundantly expressed in the anterior pituitary gland using quantitative RT-PCR and Western blotting, and demonstrated that claudin-9 is a major constituent of TJs in the FS cells of the follicles and intraglandular clefts using immunofluorescence and immunoelectron microscopy.

The follicular lumen of the anterior pituitary gland is compartmentalized by FS cells and their TJs. The barrier property of the TJs in FS cells have not been fully understood, except that the TJs of follicular FS cells in rats restrict the paracellular permeability at least for horseradish peroxidase (HRP)^44^. To understand the epithelial barrier properties of FS cells, it is important to determine the combination of claudins expressed in the tissue. In the present study, we demonstrated that the TJs of FS cells are mainly composed of claudin-9, which has been less characterized compared with other classical claudins. Since many anti-claudin antibodies recognize other claudins, we used two independent monoclonal antibodies with different epitopes and one polyclonal antibody to avoid a possible cross-reactivity problem (Fig. 3 and Supplementary Fig. S3 online). Furthermore, we demonstrated that the claudin-9 signal is closely associated with the plasma membrane of TJs using immunoelectron microscopy (Fig. 4). A previous study reported that claudin-4 is expressed in FS cells, and is localized at TJs^45^. However, we could not detect claudin-4 transcript in our RT-PCR using specific primers, which can detect claudin-4 expression in other tissues.

The TJs of FS cells are formed over one micrometer from the apical surface with occasional interruptions. Since the TJs of FS cells are unusually accompanied by an electron-dense cytoplasmic coat, part of them were initially regarded as zonula adhaerens (adherens junctions)^25^. This appearance of TJs is similar to that of tight-adherens junctions (TAJs) found between outer hair cells (OHC) and supporting Deiters cells (DC) in the inner ear^42^. The OHC-DC junctions in the inner ear are made up of two subdomains. The apical-most subdomain contains parallel claudin-14-rich TJ strands. The second subdomain is made up of an extensive anastomosing TJ strand network and a dense cytoplasmic plaque, which contains the AJ-undercoat catenin molecular complex and a massive actin cytoskeletal network in addition to ZO-1^42^. Interestingly, the second subdomain of OHC-DC junctions also contains claudin-9^40,42^, raising the possibility that claudin-9 plays a role in the formation of TJs associated with dense cytoplasmic plaques.

Since claudin-9 is specifically expressed in the FS cells in the pituitary glands and the expression is limited to a few types of epithelia, claudin-9 would be a useful marker to trace and identify FS cells. FS cells have been proposed to be the origin of some non-functioning pituitary adenomas^46^. Thus, claudin-9 could potentially be used as a diagnostic and/or prognostic marker or an immunotherapy target for subtypes of pituitary adenomas.

Although the precise roles of FS cells and their follicles are unknown, many hypotheses for their functions have been suggested. Recent reports showed that FS cells have subpopulations with different gene expression profiles, and some of them are adult pituitary stem cells, which are characterized by the expression of stem cell markers, including Sox2, S100β, Sox9, Prop1, GFRα2, and CD9^18,47–51^. Based on our immunostaining, claudin-9 appears to be expressed in almost all FS cell populations. Since the epithelial barrier function is important for stem cell behavior and their fate decision^52–54^, claudin-9-based TJs could contribute to the function of these cells through the regulation of paracellular permeability of ions, nutrients, or other specific signaling molecules. However, it is unlikely that claudin-9 is indispensable for the stem cell function of FS cells because a point mutation (F35L) in the first extracellular loop of claudin-9 in mice and a frameshift mutation at the boundary of the first transmembrane domain and the first extracellular loop of human claudin-9 (CLDN9) (P.Leu29ArgfsTer4) in human cause no obvious phenotype except for congenital hearing loss^55,56^. Since the barrier function of epithelial cells is also regulated by tTJs^35,36^, tricellulin, angulin-1, and angulin-2 might also be involved in the regulation of stemness^57^.

Another major component of TJs in FS cells is occludin. Occludin-knockout mice exhibited suckling defects in females as well as other phenotypes in the brain, stomach, bone, and testis^58^. This phenotype was speculated to be caused by increased ER stress in the mammary glands, which also express occludin^59^. It is still possible that loss of occludin and TJ functions in FS cells in the anterior pituitary gland alters the coordinated response of lactotrophs, causing the nursing defect. Nonetheless, detailed analysis of lactotrophs in claudin-9-knockout mice in the future would reveal the precise functions of FS cells and their follicles.

The functions of the pituitary follicles and FS cells have not been uncovered for more than half a century. Our observations showed that claudin-9-based TJs seal the lumen of the follicles of FS cells and it is the first step to understand their functions.

## Methods

### Animal experiments

The experiments conducted using animals strictly adhered to the compliance standards of the Japanese Guidelines for Proper Conduct of Animals Experiments and ARRIVE guidelines. The protocols for the animal experiments (approval numbers 29098, 2019023, 30112, 2020023, and 2021034) were reviewed by the Fukushima Medical University Animal Care and Use Committee and were approved by the president of the university. C57/BL6 mice used for the whole-mount staining were purchased from CLEA Japan. ICR mice and Wister rats used in the other experiments were obtained from Charles River Laboratories Japan. For the experiments using adult animals, 8–10-week-old animals were used. At least three animals including both male and female were examined for each experiment, and the data from male animals were shown unless otherwise indicated.

### Antibodies

Rat anti-occludin mAb (clone MOC37)^60^, rat anti-tricellulin mAb (clone 1E2)^61^, rat anti-angulin-1/LSR mAb^38^ and rabbit anti-angulin-2/ILDR1 pAb^38^ were previously described. Rabbit anti-occludin pAb (#LS-B2187) and rabbit anti-ZO-1 pAb (#61-7300) were purchased from Lifespan Biosciences and Thermo Fisher Scientific, respectively. Rabbit anti-S100 pAb (Z0311), rabbit anti-GH pAb (L1814), rabbit anti-LH pAb (L1827), and rabbit anti-TSH pAb (L1842) were purchased from DAKO. Goat anti-claudin-9 pAb (C-20; sc-17672), mouse anti-claudin-9 mAb (clone E-7; sc-398836), goat anti-VE-cadherin pAb (sc-6458), mouse anti-Prolactin mAb (clone H-12; sc-271773), and mouse anti-ACTH mAb (clone O2A3; sc-57018) were from Santa Cruz Biotechnology. Rabbit anti-claudin-6 pAb (#18865), rabbit anti-claudin-7 pAb (#18875), rabbit anti-claudin-8 pAb (#18885), and rabbit anti-claudin-12 pAb (#18801) were obtained from ImmunoBiological Laboratories.

Rat anti-claudin-9 mAbs were prepared with the rat iliac lymph node methods^39^. A peptide corresponding to the 16 amino acids near the carboxy terminus of mouse claudin-9 (NH_2_-Cys-ERPRGPRLGYSIPSRS-COOH; the amino-acid sequence is completely conserved in human CLDN9) was synthesized (Eurofins Genomics, Tokyo, Japan), conjugated with keyhole limpet hemocyanin (#77606; Thermo Fisher Scientific, MA,USA), mixed with Freund's complete adjuvant (F5881; Sigma-Aldrich, MO, USA) to make an emulsion and used for the immunization of two 8-week-old female Wister rats. Two weeks after immunization, the rats were euthanized and both iliac lymph nodes were aseptically harvested. The lymph nodes were minced and filtered on a 70-µm-mesh cell﻿ strainer (BD Falcon). The lymphocytes were mixed with SP2 mouse myeloma cells and fused by slowly adding 50% PEG4000 (#109727; Merck millipore, Darmstadt, Germany) in DMEM (D5796; Sigma-Aldrich) containing 4% dimethyl sulfoxide (D2650; Sigma-Aldrich). The fused cells were washed and resuspended in the hybridoma medium (78% GIT medium [#637-25715; Fujifilm-WAKO, Japan], 2% HAT supplement [#21060017; Thermo Fisher Scientific], 10% BM-CondiMed-H1 Hybridoma cloning supplement [#11088947001; Roche, Basel, Switzerland] and 10% Fetal bovine serum [F7596; Sigma-Aldrich]), and seeded in 96-well flat-bottom plates. The cells were cultured at 37 °C in a CO_2_ incubator and the hybridoma clones were examined by an enzyme-linked immunosorbent assay (ELISA) using the antigen peptide. Eight clones with a good titer were isolated, and two of them (1B1 and 1B8) were used in this study.

For the secondary antibodies used in the immunofluorescence staining, Alexa Fluor 488-conjugated donkey anti-mouse immunoglobulin G (IgG) pAb (#715-545-150), Alexa Fluor 488-conjugated donkey anti-rabbit IgG pAb (#711-545-152), Alexa Fluor 488-conjugated donkey anti-rat IgG pAb (#712-545-153), Cy3-conjugated donkey anti-mouse IgG pAb (#715-165-151), Cy3-conjugated donkey anti-rabbit IgG pAb (#711-165-152), Cy3-conjugated donkey anti-rat IgG pAb (#712-165-153), and Cy3-conjugated donkey anti-goat IgG pAb (#705-165-147) were purchased from Jackson ImmunoResearch Laboratories (USA). Alexa Fluor 647-conjugated donkey anti-goat IgG pAb (#A21447) was from Life Technologies. For immunoblotting, HRP-linked sheep anti-mouse IgG pAb (#NA931V; GE Healthcare), HRP-linked goat anti-rat IgG pAb (#NA935V; GE Healthcare), and HRP-linked rabbit anti-goat IgG pAb (#P0049; DAKO) were used. For immunoelectron microscopy, biotin-conjugated donkey anti-goat IgG pAb (#A18749; Invitrogen) and Alexa Fluor 488- and streptavidin-conjugated FluoroNanogold (#7216; Nanoprobe) were used.

### RT-PCR and qRT-PCR

Total RNAs were isolated from the pituitary glands of adult mice using TRIzol reagent (#15596026; Thermo Fisher Scientific) according to the manufacturer's instructions, and cDNA libraries were synthesized using the Primescript II 1st strand cDNA synthesis kit (#6210; Takara BIO). DNA fragments were amplified by PCR with GoTaq DNA polymerase using specific primers for each claudin, described previously^62^. The primer pairs used for amplification of angulin-1, angulin-2, and angulin-3 were 5'-CTACAACCCCTATGTGGAGTGC-3' and 5'-TAGTAGTCTCCCAGGGTCACAG-3' (angulin-1), 5'-TCTCCAAACTGGCCTGAGGA-3' and 5'-CCTGTTGTTCTTGCCTGGAG-3' (angulin-2), and 5'-AGTGCCTGACAAGAAGAAGGTG-3' and 5'-AAGACATGCCCAAGGATTCTCC-3' (angulin-3). Since the primer pairs were designed within the same exons, the DNA fragments amplified from the genome DNA served as positive controls. The images were recorded using LAS4000 (GE Healthcare), and processed with Photoshop software (Adobe, CA, USA). Real-time RT-PCR (qRT-PCR) was performed with StepOne Real-Time PCR System (Applied Biosystems, CA, USA). The RT-PCR products were used as templates for amplification using Thunderbird SYBR qPCR Mix (Toyobo, Osaka, Japan) and specific primers^62^. Relative expression levels were standardized using DNA fragments of known concentrations and normalized to the expression level of *claudin-9*. Statistical analysis was performed using Welch's t-test with Holm's correction using Excel software, and a *p* < 0.05 was considered to be statistically significant.

### Immunoprecipitation

Mouse tissues were dissected and homogenized in Lysis buffer (25 mM Tris buffer [pH 7.4], 150 mM NaCl, 2 mM EDTA, 1% Nonidet P-40, 1% cOmplete protease inhibitor cocktail [11836145001; Roche]) and incubated for 20 min with gentle rotation. The lysates were centrifuged at 12,000 × g for 30 min at 4 °C and the supernatants were pre-incubated with protein G-sepharose (#17-0618-01; GE Healthcare) to eliminate nonspecific interactions. The lysates were then incubated with protein G-sepharose coated with anti-claudin-9 mAb (clone 1B1) for 1 h at 4 °C with gentle rotation. The beads were washed five times and the bound proteins were eluted in SDS sample buffer.

### Western blotting

The cells or immunoprecipitants were mixed with SDS sample buffer and boiled for 5 min. The proteins were separated by SDS-PAGE and transferred onto a PVDF membrane. The membrane was blocked with 5% non-fat dry milk (#0646869; Morinaga Milk Industry) in Tris-buffered saline containing 0.1% Tween 20 (TBST), incubated with the primary antibody in TBST at 4 °C overnight, then HRP-conjugated secondary antibody in TBST at room temperature (RT) for 1 h, and was developed with enhanced chemiluminescence (ECL Prime; GE Healthcare). The images were recorded using LAS4000 and processed with Photoshop software.

### Immunofluorescence staining

For whole-mount staining of the P2 mouse pituitary gland, the tissue was fixed with 100% methanol at − 20 °C overnight. The sample was blocked with 2% bovine serum albumin (BSA) (A7030; Sigma-Aldrich) in PBS containing 0.1% (w/v) Triton X-100 (PBS-T) at 4 °C overnight, incubated with the primary antibody in 2% BSA in PBS-T at 4 °C overnight followed by the secondary antibody in 2% BSA in PBS-T at 4 °C overnight.

For whole-mount staining of P60 mice, the tissue was fixed with 4% paraformaldehyde (PFA) in PBS for 3 h at 4 °C, and washed with PBS at 4 °C overnight. The sample was immersed in Scale/CUBIC 1 solution (CUBIC Trial Kit; Fujifilm-WAKO, Japan) at 37 °C for 2 days, and kept in PBS-T at 4 °C overnight. The sample was then soaked in 0.1% semicarbazide hydrochloric acid solution for 1 h at RT, washed with PBS, washed with distilled water, and incubated with Immunosaver (Nisshin EM) solution at 70 °C overnight. The sample was blocked with 2% BSA in PBS-T at 4 °C overnight and primary and secondary antibody reactions were carried out in PBS containing 1% BSA and 0.5% Triton X-100 at 4 °C overnight. After washing with PBS, the sample was immersed in Scale/CUBIC 2 solution at 37 °C for 2 days.

For frozen sections, samples on coverslips were fixed with 100% methanol for 15 min at − 20 °C, blocked with 2% BSA in PBS, and treated with primary antibodies at 4 °C overnight, followed by secondary antibodies at RT for 30 min.

The samples were embedded with FLUORO-GEL II with DAPI (Electron Microscopy Sciences, USA) and observed with a fluorescence microscope (BX61; Olympus) with a 10 × objective lens (UPlanSApo 10x; Olympus) equipped with a mercury lump, dichroic filter set (NIBA), and a cooled charge-coupled device (CCD) camera (DP71; Olympus) or a laser scanning confocal microscope (FV1000; Olympus) with 10 × objective lens (UPlanSApo 10x; Olympus) or 60 × oil-immersion objective lens (UPlanSApo 60x; Olympus) at laser wavelengths of 405, 488, and 559 nm. The images were acquired with cellSens ver. 1.14 (Olympus) or fluoview ver. 4.2b (Olympus) and processed with ImageJ and Photoshop.

### Immunoelectron microscopy

The adult mouse pituitary gland was dissected and fixed with 50 mM HEPES–NaOH (pH 7.4) containing 150 mM NaCl, 2% PFA, 0.002% glutaraldehyde (GA), and 5% sucrose for 2 h at RT, cryoprotected with 30% sucrose and embedded in Tissue-Tek OCT compound (Sakura Finetek, Japan). The sample was quickly frozen in liquid nitrogen and cut into 8 µm-thick sections in a cryostat at − 20 °C. The sections were soaked in 0.1% semicarbazide hydrochloric acid solution for 1 h at RT, washed with PBS, washed with distilled water, and soaked in Immunosaver solution at 70 °C overnight. The sections were permeabilized with PBS-T for 20 min at RT, blocked with 2% BSA in PBS, and treated with goat anti-claudin-9 pAb in PBS containing 2% BSA at 4 °C overnight. The sections were then treated with biotin-conjugated secondary antibody, followed by streptavidin conjugated-FluoroNanogold in PBS containing 2% BSA for 1 h at RT. After fixation with 1% GA for 30 min at RT, gold enhancement was performed using GOLDENHANCE EM (Nanoprobes) as per the manufacturer’s instruction. The samples were postfixed with 0.5% OsO4 in 0.1 M phosphate buffer for 15 min at 4 °C, followed by dehydration in an ethanol series (65%, 75%, 85%, 95%, 99% and 100%) and propylene oxide and embedding in Epon 812 resin. Ultrathin sections of ~ 70-nm thickness were stained with 1% hafnium chloride in methanol and lead citrate, and then observed under an electron microscope (JEM-1011; JEOL).

### Immunohistochemistry

FFPE samples of normal human pituitary gland tissue array (#PIT501) were purchased from US Biomax (Darwood, MD, USA). The sections of HEK293T cells expressing human CLDN9 were prepared as described previously^62^. Paraffinized samples were treated with 0.3% hydrogen peroxide-containing methanol at RT for 20 min to inactivate the endogenous peroxidase. Then, heat-mediated epitope retrieval was conducted using Immunosaver according to the manufacturer's instructions. Subsequently, the sections were blocked with 5% non-fat dry milk and incubated with the primary antibody at 4 °C overnight. The secondary antibody incubation was carried out at RT for 30 min. Samples were developed with 3,3'-diaminobenzidine (DAB) solution (50 mM Tris buffer [pH 7.5], 0.02% DAB, 0.005% hydrogen peroxide) and counter-stained with hematoxylin. Dehydrated samples were embedded and imaged using a DP controller (Olympus, Tokyo, Japan).

## Supplementary Information

Supplementary Information.

## Data Availability

The datasets of the current study are available from the corresponding author on reasonable request.
