# Supplementary material for: Claudin-9 constitutes tight junctions of folliculo-stellate cells in the anterior pituitary gland
Source: Sci Rep. 2021 Nov 4;11:21642. doi: 10.1038/s41598-021-01004-z (PMC8568902; doi:10.1038/s41598-021-01004-z)
Supplement: Supplementary file 1 — Supplementary Information. [file 41598_2021_1004_MOESM1_ESM.pdf]

Supplementary information:

Claudin-9 constitutes tight junctions of folliculo-stellate cells in the anterior pituitary gland.

Atsuko Y Higashi[1,2], Tomohito Higashi[1*], Kyoko Furuse[3], Kana Ozeki[1], Mikio Furuse[3,4], Hideki Chiba[1]

[1]Department of Basic Pathology, Fukushima Medical University, Fukushima 960-1295, Japan.
[2]Department of Nephrology and Hypertension, Fukushima Medical University, Fukushima 960-1295, Japan.
[3]Division of Cell Structure, National Institute for Physiological Sciences, Okazaki, Aichi 444-8787, Japan.
[4]Department of Physiological Sciences, School of Life Science, SOKENDAI (Graduate University for Advanced Studies), Okazaki, Aichi 444-8585, Japan.

*Address correspondence to:
Tomohito Higashi, Ph.D. (ORCID ID: 0000-0001-5616-1477)
Associate Professor
Department of Basic Pathology, Fukushima Medical University
1 Hikariga-oka, Fukushima, 960-1295, Japan.
tohigash@fmu.ac.jp
+81.24.547.1169

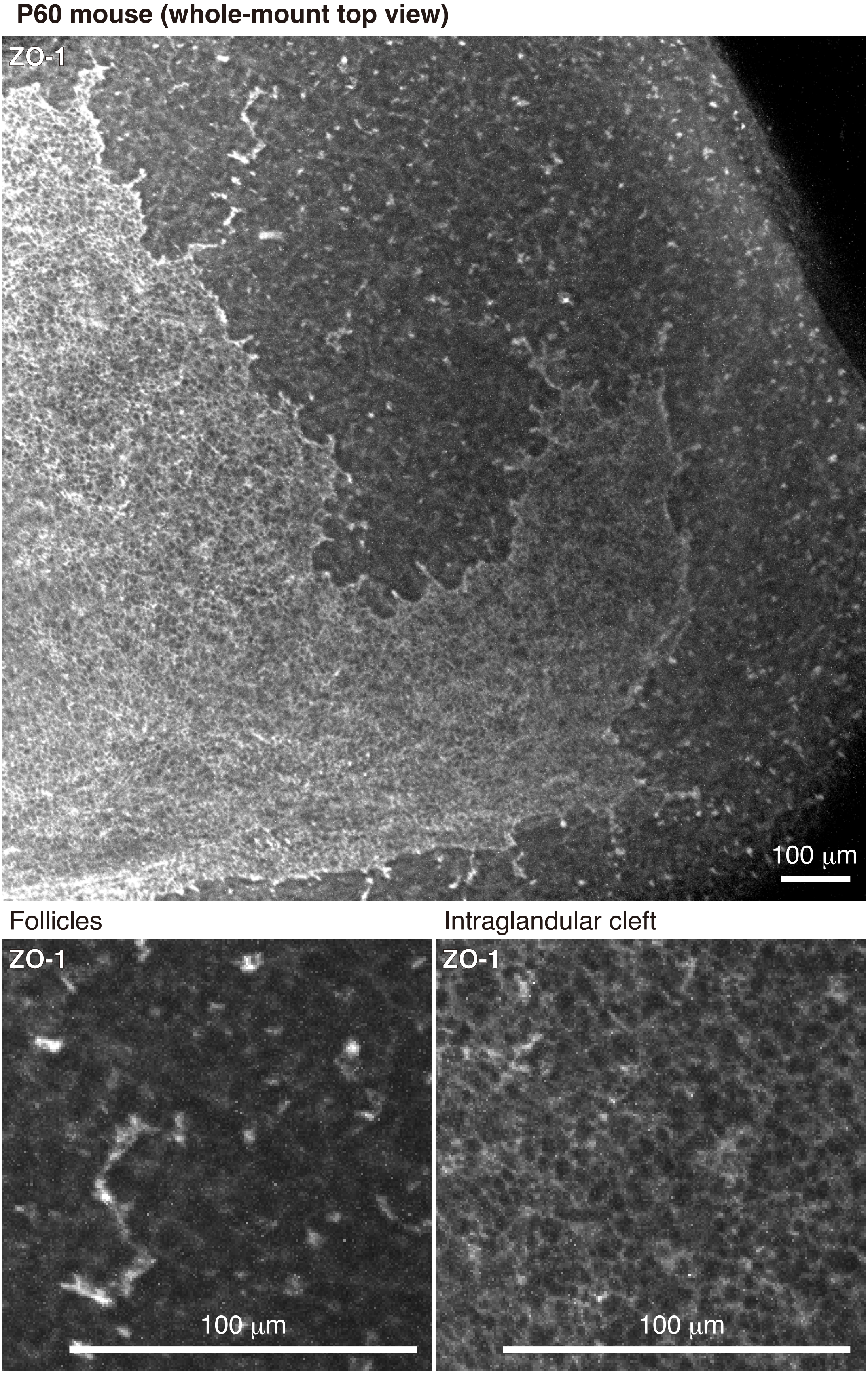

Figure S1. Organization of FS cell lumens in the adult pituitary gland.
Whole-mount staining of the pituitary gland of a P60 mouse using anti-ZO-1 pAb. Note that the small follicles have independent lumens and they are uniformly distributed throughout the tissue.

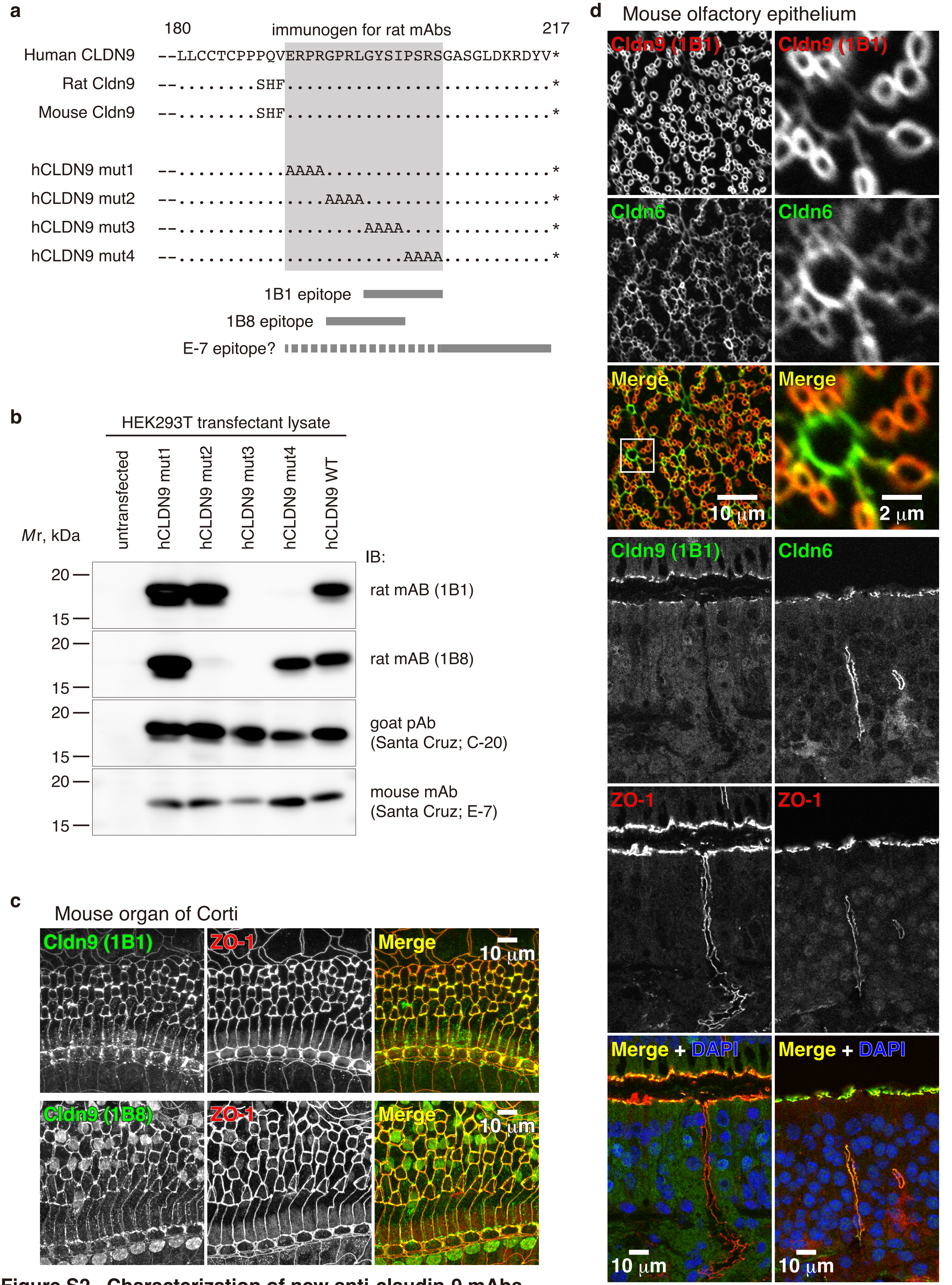

Figure S2. Characterization of new anti-claudin-9 mAbs.
a. Amino-acid sequences of claudin-9. **b.** Immunoblotting of HEK293T lysates expressing wild-type (WT) and mutant claudin-9. Note that the epitopes of clones 1B1 and 1B8 are different. **c.** Immunostaining of the adult mouse sensory epithelium in the inner ear. **d.** Immunostaining of the adult mouse olfactory epithelium.

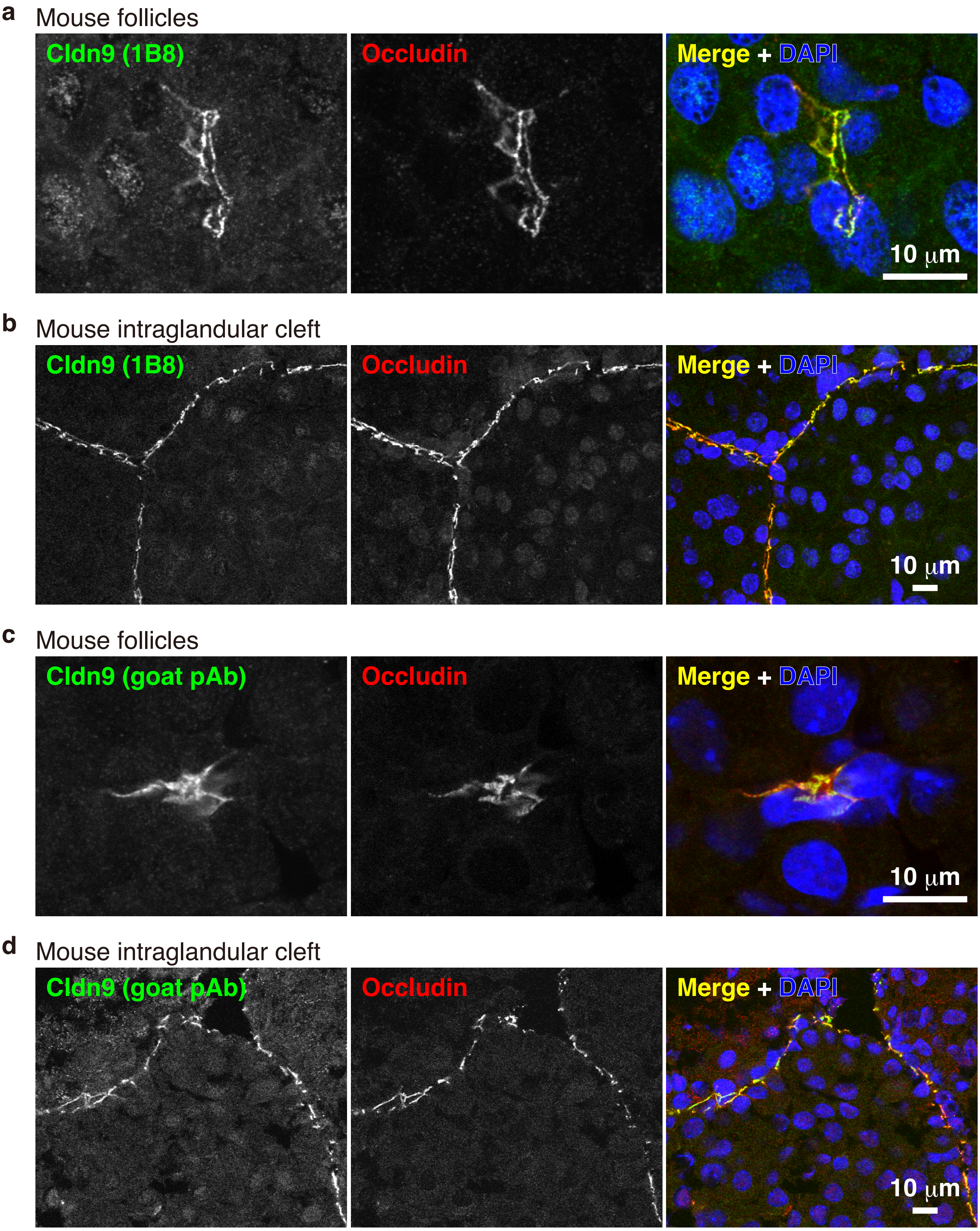

Figure S3. Immunostaining of the FS cells using anti-claudin-9 mAb (clone 1B8) and goat pAb. The adult mouse anterior pituitary gland was stained with rat anti-claudin-9 mAb clone 1B8 (**a** and **b**) or goat anti-claudin-9 pAb (**c** and **d**) (green), anti-occludin pAb (red), and DAPI (blue). Note that cell-cell junctions between the FS cells in both follicles (**a** and **c**) and intraglandular clefts (**b** and **d**) have signals similar to those stained with the rat anti-claudin-9 (clone 1B1).

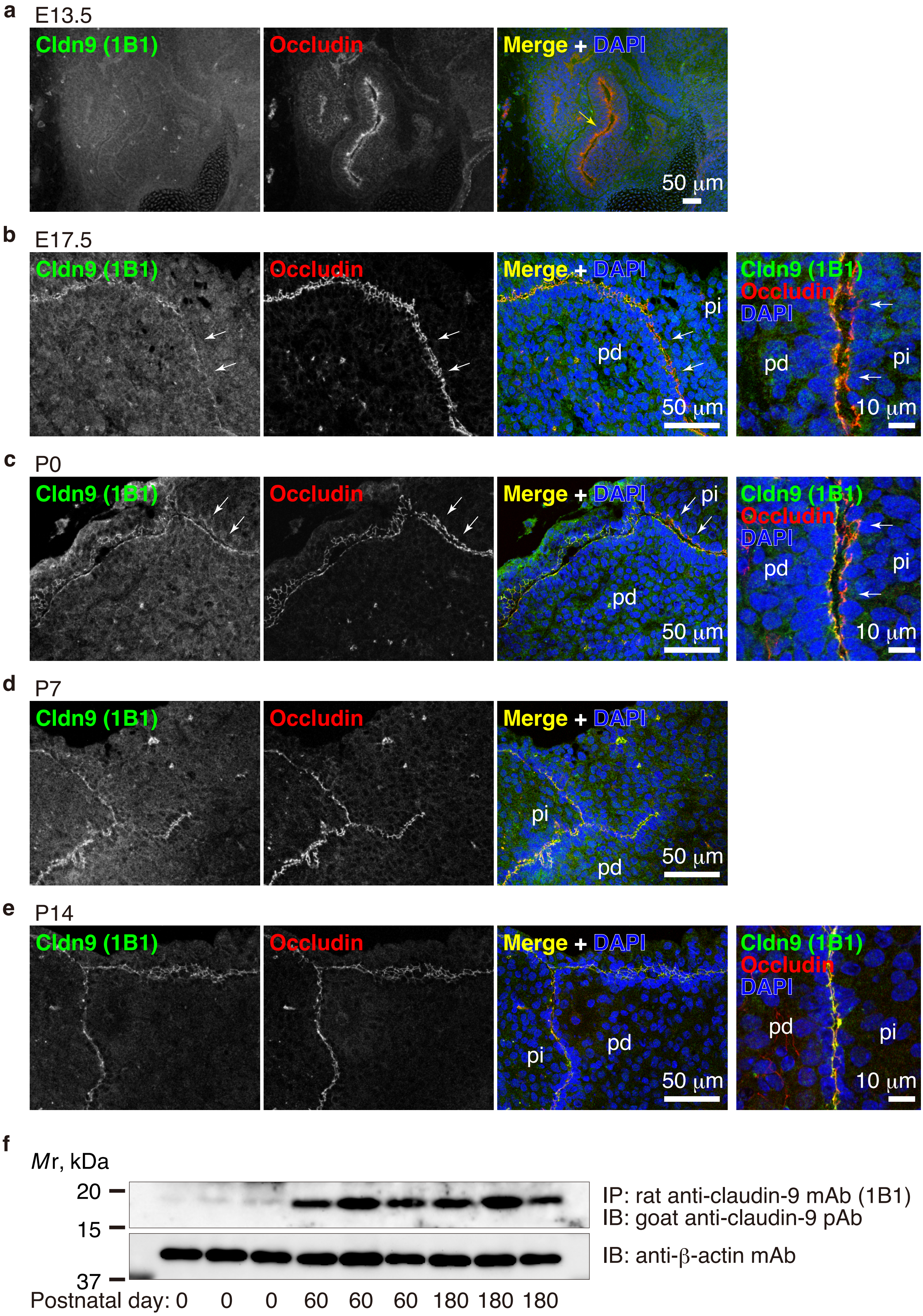

Figure S4. Expression pattern of claudin-9 in each developmental stage of the anterior pituitary gland. **a**-**e**, Immunostaining of embryos (E13.5 [**a**] and E17.5 [**b**]) and neonates (P0 [**c**], P7 [**d**], and P14 [**e**]) using anti-claudin-9 mAb (Clone 1B1) (green), anti-occludin pAb (red) and DAPI. Blow-up images of the intraglandular cleft between the pars intermedia (pi) and pars distalis (pd) are shown (right). Note that claudin-9 is not expressed in Rathke's pouch (yellow arrow in **a**) and the FS cells in the intraglandular cleft at the pars intermedia side (white arrows in **b** and **c**) at E17.5 and P0. **f**, Immunoblotting of pituitary glands. Note that claudin-9 expression is increased after birth.

Fig. 3a

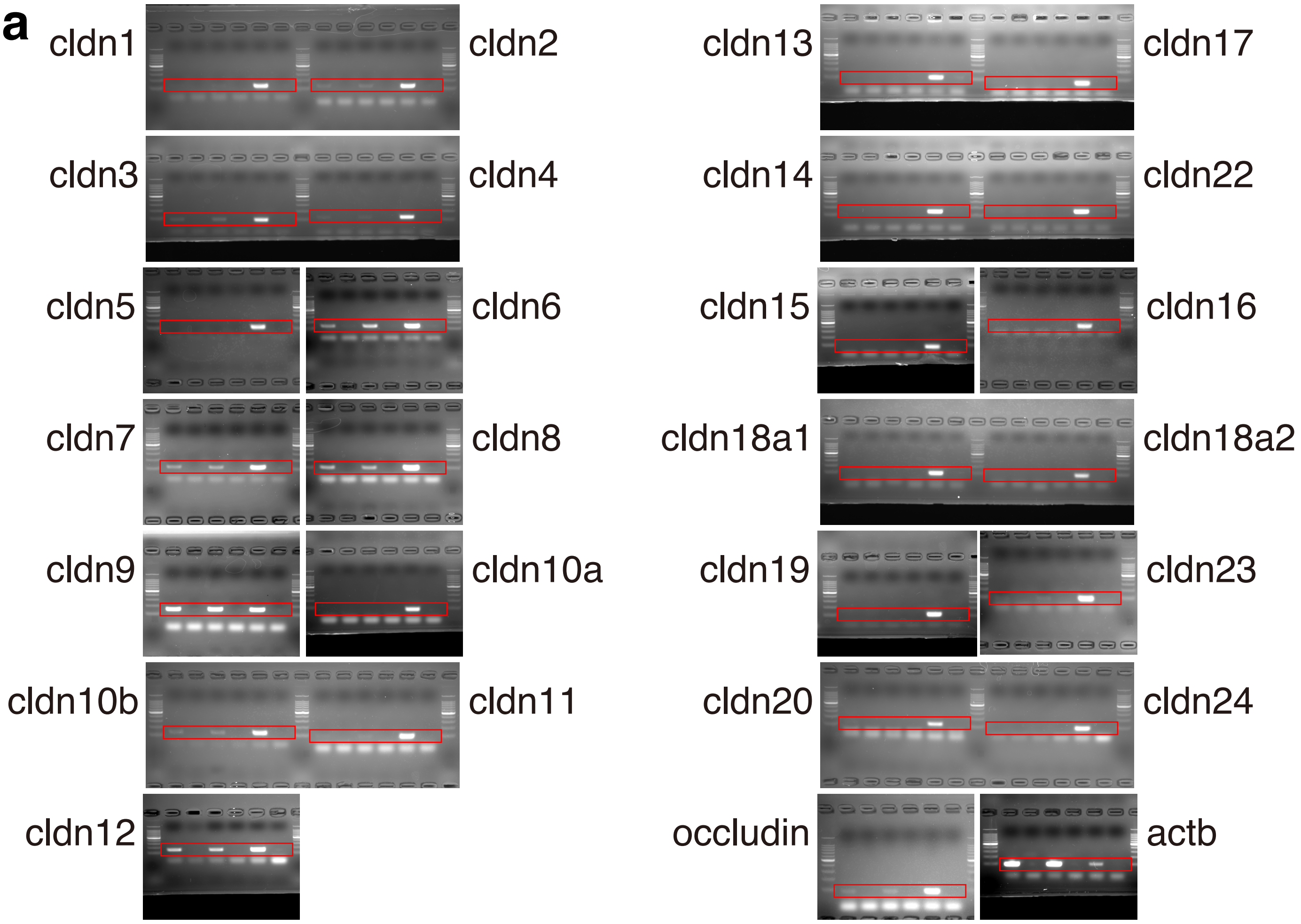

Fig. 5a

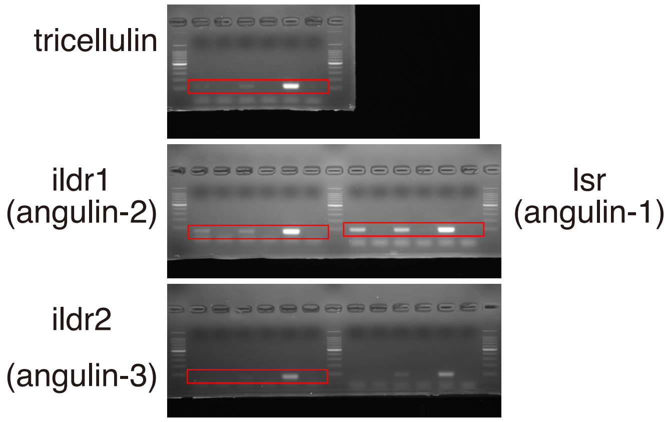

Fig. S4f

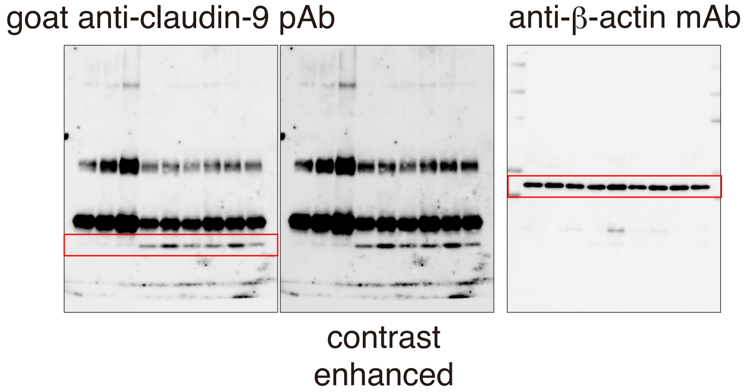

Fig. 3c

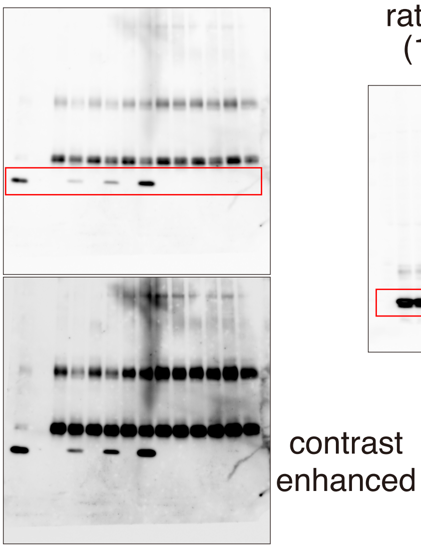

Fig. S2b

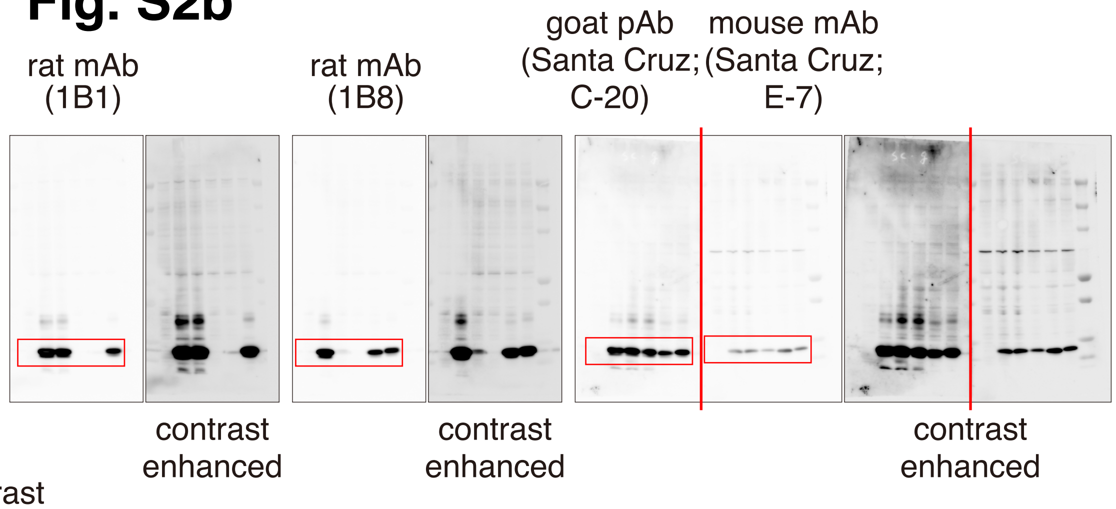

Uncropped gel images
